# Identification of an Aberrant Transligamentous Branch of the Median Nerve during Carpal Tunnel Release using Ultrasound Guidance

**DOI:** 10.1016/j.jhsg.2025.02.008

**Published:** 2025-03-21

**Authors:** Douglas Hoffman, Jay Smith, Katelyn Henningsen

**Affiliations:** ∗ Departments of Orthopedics and Radiology, Essentia Health, Duluth, MN; † Sonex Health, Eagan, MN; ‡ University of Minnesota Medical School, Duluth, MN

**Keywords:** Carpal tunnel, Median nerve, Ultrasound

## Abstract

Ultrasound is a well-established tool for diagnosing carpal tunnel syndrome and guiding therapeutic release procedures. Variations in carpal tunnel anatomy, including the palmar cutaneous and thenar motor branches, are well-documented. This case describes a novel transligamentous branch of the median nerve, resembling a second palmar cutaneous branch, identified with diagnostic ultrasound prior to an ultrasound-guided carpal tunnel release. Continuous visualization of this aberrant transligamentous branch during the ultrasound-guided carpal tunnel release procedure enabled precise avoidance, reducing the risk of iatrogenic injury.

Carpal tunnel syndrome is the most common compressive neuropathy of the upper extremity with the majority of cases having an idiopathic etiology. The median nerve (MN) is known to have many variations as it traverses through the carpal tunnel, some of which may be at risk of iatrogenic injury during a carpal tunnel release (CTR).^1^ The use of ultrasound to diagnose carpal tunnel syndrome (CTS) by documenting median nerve enlargement has been validated in multiple studies.^2^, ^3^, ^4^ Furthermore, ultrasound-guided CTR has been shown to be a safe and effective alternative to the traditional techniques of open or endoscopic CTR.^5^^,^^6^ Compared to open CTR and endoscopic CTR, ultrasound-guided CTR provides a greater field of view of the carpal tunnel structures, with the added advantage of being able to identify anatomic variants within the carpal tunnel that may be at risk of iatrogenic injury. This case demonstrates how a diagnostic US prior to performing an ultrasound-guided CTR identified a transligamentous branch (TLB) of the MN and the subsequent ability to preserve its integrity during a ultrasound-guided CTR.

## Case Study

An 80 year-old, right-hand dominant female presented with unilateral left hand nocturnal paresthesias that progressed to continuous numbness involving the thumb, index, long, and ring fingers over the prior three months. Both the past medical history and surgical history were noncontributory. She was referred for a diagnostic ultrasound and consultation for a ultrasound-guided CTR. No prior work-up had been performed.

A focused physical examination showed left thenar muscle atrophy as well as a bony prominence of the first carpometacarpal joint with mild tenderness to palpation. Two point discrimination of fine touch was >10 mm for the distal volar pads of the left thumb, index, and long fingers, and 6 mm of the small finger. Palmar thumb abduction strength was 4/5. There was a positive carpal compression test, negative Tinel’s sign over the carpal tunnel, and a positive Phalen’s test.

**Figure 1:**
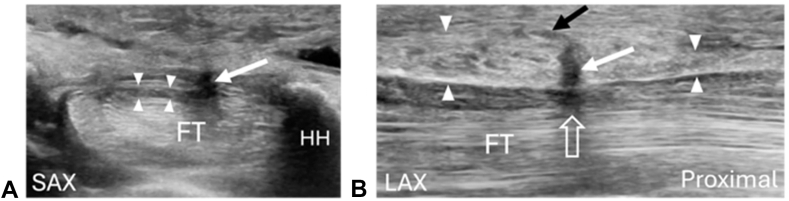
Prerelease images. Images of the carpal tunnel prior to the ultrasound-guided CTR. **A** Short axis (SAX) or anatomic transverse image of the midcarpal tunnel at the level of the hook of the hamate (HH) showing a hypoechoic and flattened MN (arrowheads) and a hypoechoic appearing TLB (arrow) arising from the ulnar side of the MN and traversing through the TCL complex and into the subcutaneous tissue. The TLB is represented by the vertical shadow, similar to the ultrasound appearance of a TMB. Note that the TLB of the MN is on the ulnar aspect of the nerve which potentially increases the risk of iatrogenic injury during a CTR. FT, flexor tendons. Left = radial, Top = superficial. **B** Long axis (LAX) or anatomic sagittal image of the MN within the CT showing narrowing (i.e., “hourglass deformity”) of the MN within the midcarpal tunnel (open arrow) and the TLB arising from the MN and passing through the TCL complex (white arrow) and into the subcutaneous tissue (black arrow). The TCL complex is outlined by the arrowheads.

A diagnostic ultrasound of the carpal tunnel was performed using a linear array 4–18 MHz transducer. Examination revealed an enlarged MN of 13 mm^2^ (upper limit of normal 10 mm^2^) in the proximal carpal tunnel region accompanied by reduced echogenicity (ie, hypoechoic) and a loss of the normal fascicular echotexture. The MN was notably narrowed within the midcarpal tunnel (Fig. 1A, B). Both the palmar cutaneous (PCB) and the thenar motor branches (TMB) of the MN appeared normal. The PCB was visualized as a single fascicle nerve that branched from the radial aspect of the MN several centimeters proximal to the carpal tunnel and penetrated the distal forearm fascia ulnar to the flexor carpi radialis tendon to traverse into the palmar subcutaneous tissue. The thenar motor branch originated from the radial aspect of the MN distal to the transverse carpal ligament (TCL) complex and traversed into the thenar muscle group. In addition, a TLB of the MN was identified and branched from the ulnar aspect of the MN within the midcarpal tunnel, traversed through the TCL complex and coursed superficially into the palmar subcutaneous tissue, consistent with a second PCB of the MN (Fig, 1A, B).

**Figure 2:**
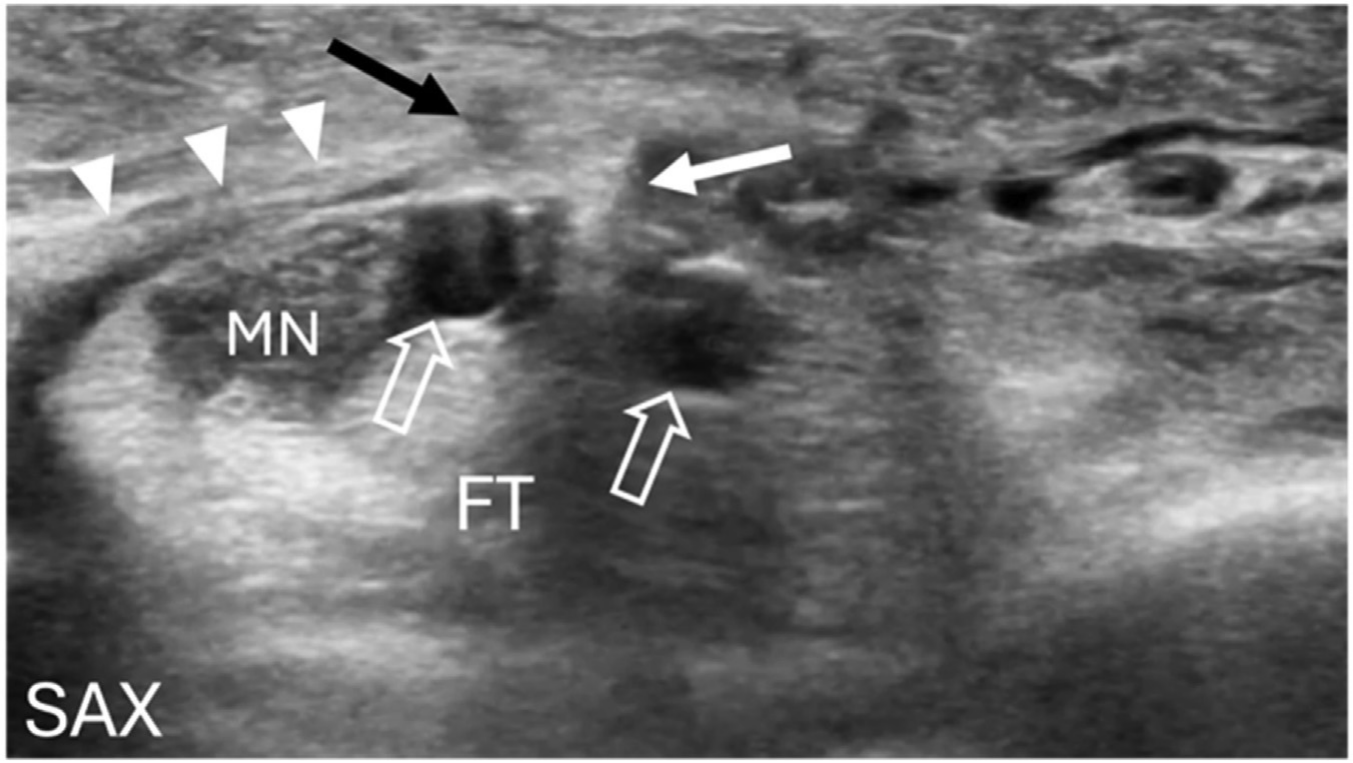
Transection of the transverse carpal ligament adjacent to the TLB of the MN. Short-axis or anatomic transverse US image during ultrasound-guided CTR. The device has been positioned in the carpal tunnel, ulnar to the MN. Following balloon deployment (open arrows) and confirmation of accurate positioning relative to the surrounding structures, the retrograde cutting blade (white arrow) has been activated from its recessed position. The cutting blade is shown adjacent and ulnar to the TLB (black arrow) during the retrograde cut of the TCL complex (arrowheads). FT = flexor tendons. Left = radial, Top = superficial.

The patient elected to undergo an office-based left ultrasound-guided CTR under local anesthesia using a linear array 4–18 MHz transducer. Local anesthetic was administered into the distal volar forearm subcutaneous tissue as well as into the carpal tunnel, while also performing a hydrodissection of the MN to mobilize it from the TCL complex. Using ultrasound guidance, a #15 scalpel was used to create a 4 mm longitudinal incision at the proximal wrist crease, approximately midline between the ulnar aspect of the MN and the ulnar artery (UA). Under US guidance, a Penfield dilator was inserted into the carpal tunnel to mobilize hypertrophic subsynovial tissue from the undersurface of the TCL complex. A device specifically designed for ultrasound-guided CTR (UltraglideCTR, Sonex Health, Eagan, MN) was then inserted and advanced under ultrasound guidance into the carpal tunnel between the MN and UA until the device tip was positioned in the region of the distal TCL complex. The device balloons, located on each side of the cutting blade, were then inflated to create space between the MN and the cutting blade as well as to improve visualization of the adjacent anatomy. The position of the device tip was next assessed in relation to the MN, UA, the crossover of the third common digital nerve, and the superficial palmar arch, and confirmed to be in an appropriate location to proceed with a retrograde transection of the TCL. In addition, the TLB of the MN was identified and the anticipated path of the retrograde cut was confirmed to be ulnar to the TLB. The TCL complex was subsequently transected in a retrograde fashion by visualizing the cutting blade in a short-axis (anatomic transverse) probe orientation while slowly “inching” both the US transducer and cutting blade proximally. Before cutting the TCL at the level of the TLB, the TLB was first visualized with ultrasound and the cutting blade was carefully advanced proximally past the TLB to confirm its position on the ulnar side of the TLB (Fig. 2). Once the transection of the TCL complex was completed, the cutting blade was placed in its recessed position, the balloons deflated, and the CTR device was used to probe the TCL complex and confirm a complete transection before removal. Post-release images confirmed an intact TLB. The incision was closed with adhesive strips and a compression dressing applied.

**Figure 3:**
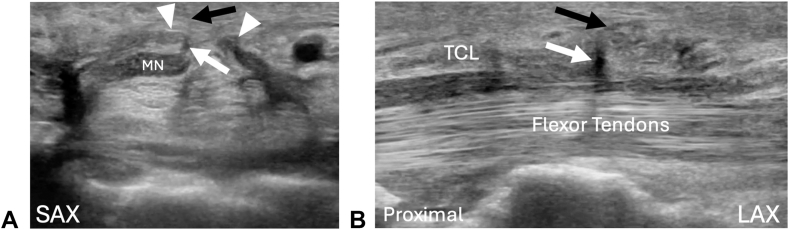
Six week follow-up ultrasound. Short-axis or anatomic transverse and LAX ultrasound or anatomic sagittal images showing a released TCL complex and an intact TLB of the MN. **A** SAX image at the midcarpal tunnel (comparable to Figs. 1A and 2) showing transection of the TCL complex (white arrowheads). Note the palmar or superficial position of the MN relative to Figure 1A. Once again, the TLB is seen branching from the ulnar side of the MN (white arrow) and traversing into the subcutaneous tissue (black arrow). **B** LAX view of the carpal tunnel (comparable to Fig. 1B) showing an intact TLB branching from the MN in the midcarpal tunnel (white arrow) and traversing through the TCL complex and into the subcutaneous tissue (black arrow).

The patient was seen 1 week after surgery with near complete resolution of hand paresthesias. At the second follow up visit 6 weeks later there was complete resolution of all CTS symptoms. A follow-up ultrasound confirmed an intact TLB (Fig. 3). At that time, she gave her consent for the creation of this case report.

## Discussion

Ultrasound-guided CTR is an alternative to open or endoscopic techniques and is commonly performed as a wide-awake, office-based procedure. Traditionally, the history and physical examination combined with electrodiagnostic studies have been commonly used to diagnose CTS. However, ultrasound has been validated as a method for assisting in the diagnosis of CTS with a comparable sensitivity and higher specificity to electrodiagnostic studies.^4^ Additional advantages of ultrasound include comfort, reduced cost, direct visualization of the carpal tunnel anatomy, and potentially greater accessibility.^3^^,^^4^ Sonographic examination of the carpal tunnel can also identify most secondary causes of CTS as well as anatomic variants that may influence treatment approaches and surgical planning.^2^^,^^4^^,^^7^^,^^8^

**Figure 4:**
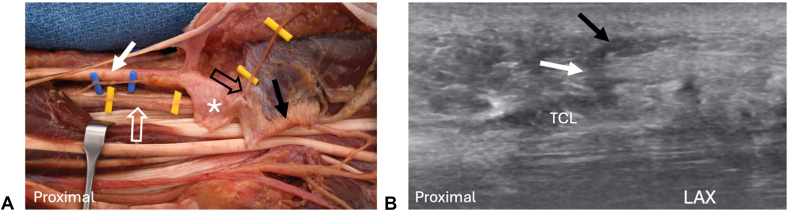
Cadaveric dissection and associated ultrasound image of an unembalmed anatomic specimen with a transligamentous palmar cutaneous branch of the median nerve similar to the current case. The transligamentous palmar cutaneous branch of the median nerve was detected and characterized with US prior to the dissection. **A** Dissection of the carpal tunnel in which a second PCB of the MN (cut yellow vascular loops) was present. Unlike the current case, in this specimen the second transligamentous PCB arose from the MN proximal to the carpal tunnel and passed separately into the carpal tunnel before penetrating the TCL complex in the proximal carpal tunnel region. The specimen was subsequently dissected to confirm the ultrasound findings. The MN (white open arrow) passes into the carpal tunnel, first deep to the volar antebrachial fascia (asterisk), and then under the transected TCL complex (black arrow). The PCB of the MN (cut blue vascular loops) has a normal course, branching from the MN in the distal forearm region, traversing on the radial side of the MN and adjacent to the flexor carpi radialis tendon (FCR, white arrow), and passing into the subcutaneous tissue. A second PCB arises from the MN (cut yellow vascular loops) travels on the palmar side of the MN, deep to the volar antebrachial fascia, and passes into the carpal tunnel and subsequently through the proximal edge of the TCL complex (black open arrow), and into the subcutaneous tissue consistent with a second transligamentous PCB, as seen in the current case. **B** LAX view of the carpal tunnel (comparable to Fig. 3B) showing the second PCB of the MN traversing through the TCL complex (white arrow) and into the palmar subcutaneous tissue (black arrow). Note the similar appearance of the TLB of the MN in the cadaveric dissection to the TLB of the MN in our case (Fig. 3B).

It has been established that ultrasound can identify both the PCB and TMB of the MN, including anatomic anomalies of both nerve branches.^8^ It is also well established that the TMB can have a transligamentous course with the reported incidence ranging from 3.3% to 42.3%.^9^ In the current case, the thenar motor branch followed a standard anatomical route, with a single branch originating from the MN distal to the TCL complex (i.e., extraligamentous course). The presence of other types of TLBs has also been reported. Beckman et al^7^ described a TLB of the MN that originated in the carpal tunnel, traversed through the TCL complex, and then anastomosed with the first common palmar digital nerve. However, to our knowledge, this is the first published report demonstrating the identification of a TLB of the MN that is consistent with a second PCB of the MN. In the majority of cases, the PCB branches from the MN within the distal forearm region, traverses adjacent to the ulnar side of the flexor carpi radialis tendon, and terminates in the palmar subcutaneous tissue as a purely sensory nerve. However, variations in PCB of the MN are common, including multiple branches.^1^^,^^8^^,^^10^ The TLB of the MN in this case branched from the MN midway through the carpal tunnel and traversed through the TCL complex before terminating in the subcutaneous tissue in the midpalm region. It should be noted that the origin of the TLB was on the ulnar side of the MN which would potentially increase the risk of iatrogenic injury since the standard approach to transection of the TCL complex is directly above or ulnar to the MN to avoid injury to the PCB. In this case, the identification and careful ultrasound mapping of the TLB through the TCL complex at the time of the initial diagnostic ultrasound allowed surgical planning to avoid iatrogenic injury. As shown in Figure 4, one of the coauthors encountered a similar TLB while scanning an unembalmed cadaveric dissection. Subsequent dissection confirmed the presence of a TLB of the MN as a second PCB and corroborates the presence of the novel TLB of the MN described in this case study (Fig. 4).

In summary, the sonographic identification of a TLB prior to performing an ultrasound-guided CTR allowed presurgical planning and careful positioning of the CTR device prior to TCL complex transection in order to avoid iatrogenic injury. As diagnostic ultrasound and ultrasound-guided CTR become more commonplace, knowledge of anatomic variants within the CT will potentially avoid iatrogenic injury during a CTR.

## Conflicts of Interest

Dr Smith reports the following: Chief Medical Officer and employee at Sonex Health; stock/stock options, Sonex Health; and royalties/license agreement, Sonex Health. Dr Hoffman reports being a paid consultant for Sonex Health and Samsung – Neurologica. No benefits in any form have been received or will be received by the other author related directly to this article.
